# Hypothalamic Control of Liver Health and Disease: From Circuits to Pathophysiology and Therapies

**DOI:** 10.1002/advs.76747

**Published:** 2026-07-28

**Authors:** Qin Tang, Jiahui Li, Haiying Song, Ailin Zhang, Haifeng Zeng, Yining Xu, Li Mo, Jinhan He

**Affiliations:** ^1^ Department of Pharmacy Institute of Clinical Pharmacy West China Hospital of Sichuan University Chengdu Sichuan China; ^2^ West China School of Pharmacy Sichuan University Chengdu Sichuan China; ^3^ Health Management Center of West China Hospital Sichuan University Chengdu Sichuan China

**Keywords:** hypothalamus–liver axis, liver pathophysiology, neural circuits, therapeutic strategies

## Abstract

The liver is the body's principal metabolic hub, reprogramming its functions according to physiological demands. Beyond local nutrient and hormonal cues, the hypothalamus acts as a central command node, integrating systemic signals to coordinate hepatic glucose and lipid homeostasis, bile acid flux, and regeneration. Dysregulation of this hypothalamus–liver axis is a core driver of liver disease, from steatotic liver disease to hepatocellular carcinoma. In this Review, we discuss established knowledge and recent advances in the neural and neuroendocrine pathways that connect the hypothalamus to the liver. We examine how specific signals guide physiological adaptation and how their maladaptive plasticity fuels disease progression. We also evaluate emerging therapeutic strategies that target this neuro‐hepatic interface, including circuit‐specific pharmacology and bioelectronic modulation, and outline key translational challenges. Decoding the hypothalamus–liver axis not only reveals a fundamental principle of inter‐organ communication but also points to new approaches for restoring liver homeostasis.

## Graphical abstract

This review delineates the hypothalamic circuits that control liver homeostasis via autonomic and neuroendocrine pathways. Dysregulation of this hypothalamus–liver axis drives disease progression across a spectrum including steatotic liver disease, liver inflammation and injury, fibrosis, cirrhosis, and hepatocellular carcinoma. Targeted therapies, including circuit‐specific pharmacology and bioelectronic modulation, offer new treatment horizons.

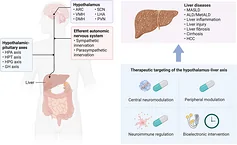

## 1 Introduction

The liver is the principal homeostatic organ for systemic metabolism [1]. It coordinates essential processes including glucose, lipid, and amino acid metabolism, plasma protein synthesis, xenobiotic detoxification, and immunomodulation via the concerted activity of multiple hepatic cell types [1]. Acting as a dynamic nutrient buffer, the liver stores glucose as glycogen postprandially and releases it via glycogenolysis and gluconeogenesis during fasting, while also partitioning lipids between oxidation, storage, and export [1]. Hepatic metabolism is under continuous top‐down regulation by the central nervous system. In particular, the hypothalamus provides rapid neural and chronic neuroendocrine pathways that adapt liver function to whole‐body energetic and physiological demands.

As the brain's master metabolic integrator, the hypothalamus receives and interprets peripheral, environmental and neural signals to maintain systemic energy homeostasis [2, 3]. The arcuate nucleus (ARC), positioned adjacent to the permeable median eminence, acts as a primary sensor of circulating factors such as leptin and insulin. It contains key first‐order neurons, including orexigenic neuropeptide Y/agouti‐related protein (NPY/AgRP) and anorexigenic pro‐opiomelanocortin (POMC) cells [3, 4]. Other hypothalamic regions, including the ventromedial (VMH), dorsomedial (DMH), lateral hypothalamic area (LHA), and suprachiasmatic nucleus (SCN), further integrate these signals with inputs from other brain areas [5, 6]. The paraventricular nucleus (PVN) serves as the principal output hub to coordinate two major effector pathways: rapid autonomic adjustments via sympathetic and parasympathetic outflow to the liver, and sustained endocrine modulation through hypothalamic‐pituitary axes [2, 5]. Through these dual channels, the hypothalamus exerts hierarchical control over hepatic metabolism.

Thus, the hypothalamus and liver engage in continuous, bidirectional communication. The liver provides metabolic feedback to hypothalamic sensing mechanisms, while the hypothalamus dynamically adjusts hepatic function through integrated neural and hormonal commands [7]. This hypothalamus–liver axis is essential for maintaining hepatic homeostasis. Its dysregulation is increasingly implicated in the pathogenesis of type 2 diabetes, metabolic dysfunction‐associated steatotic liver disease (MASLD), alcohol‐related liver disease (ALD) and the emerging category of metabolic dysfunction and alcohol‐associated liver disease (MetALD), as well as the broader spectrum of liver pathology, including inflammation, injury, fibrosis, cirrhosis and hepatocellular carcinoma (HCC).

In this Review, we focus on the hypothalamus‐liver axis as a critical regulator of hepatic physiology and pathology. We first outline its anatomical and functional organization, including direct autonomic innervation and indirect hypothalamic‐pituitary pathways. We then examine its role in physiological hepatic control before detailing how disrupted signaling contributes to disease initiation and progression. Finally, we evaluate emerging therapeutic strategies targeting this neuro‐hepatic interface, highlight translational challenges, and propose future research directions.

## 2 Neural and Neuroendocrine Pathways From the Hypothalamus to the Liver

The hypothalamus governs the liver through autonomic and neuroendocrine pathways [8]. This section details the anatomical and functional architecture of these circuits, beginning with direct autonomic neural circuits that mediate rapid hypothalamic control, followed by neuroendocrine pathways via hypothalamic‐pituitary axes that orchestrate slower, hormonally driven adaptations.

### 2.1 Autonomic Innervation of the Liver

The liver receives dual autonomic input via sympathetic and parasympathetic branches, forming a multi‐synaptic circuit that originates in the hypothalamus [8, 9].

#### 2.1.1 Sympathetic Innervation

**FIGURE 1:**
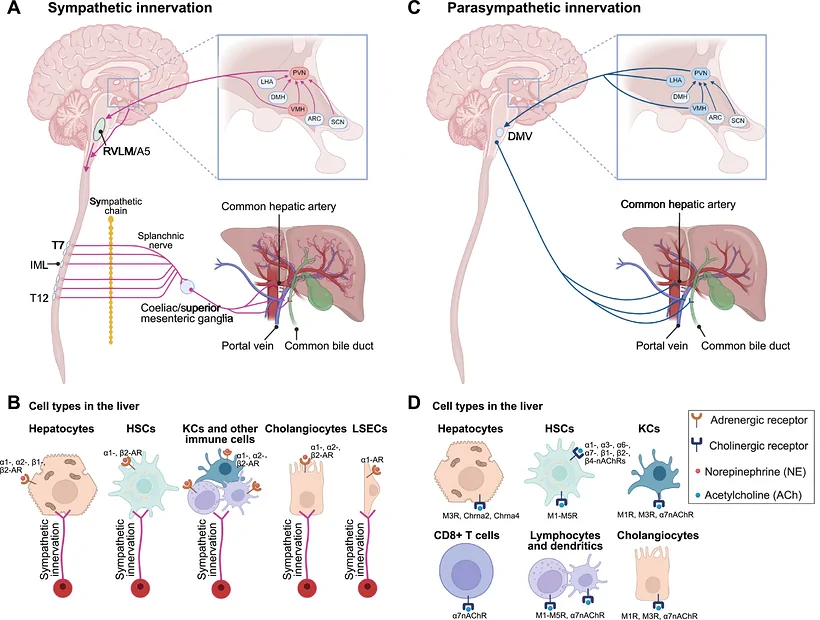
Autonomic innervation of the liver. (A) Schematic illustration of sympathetic pathways from the hypothalamus to the liver. (B) Adrenergic receptor distribution on hepatic cell types. (C) Schematic illustration of the parasympathetic pathways from the hypothalamus to the liver. (D) Muscarinic and nicotinic receptor distribution on hepatic cell types. ARC, arcuate nucleus; DMH, dorsomedial hypothalamus; HSC, Hepatic stellate cells; IML, intermediolateral column; KCs, Kupffer cells; LHA, lateral hypothalamic area; LSEC, liver sinusoidal endothelial cells; PVN, paraventricular nucleus; RVLM, rostral ventrolateral medulla; SCN, suprachiasmatic nucleus; VMH, ventromedial hypothalamus. Created in BioRender. Tang, Q. (2026) https://BioRender.com/bnfaatr.

Sympathetic innervation originates from premotor neurons in hypothalamic nuclei such as the PVN and VMH [10, 11]. These neurons project—directly or indirectly via brainstem relay centers, including the rostral ventrolateral medulla, the A5 region, and the para‐pyramidal area—to sympathetic preganglionic neurons located in the spinal cord intermediolateral column (T7‐T12 segments) [8, 12]. Peripheral outflow follows a two‐neuron chain: preganglionic fibers travel via the splanchnic nerves and synapse in the celiac and superior mesenteric ganglia, and postganglionic fibers innervate the hepatic hilum accompanying the major hepatic vessels [13] (Figure 1A).

Sympathetic innervation initiates during late embryogenesis and increases postnatally [14]. Fibers consistently form plexuses along the portal triad structures, with notable species heterogeneity in parenchymal penetration [13, 14].

Neurochemically, sympathetic terminals primarily release norepinephrine (NE), which acts on adrenergic receptors (ARs) expressed on various liver cell types (Figure 1B). Hepatocytes predominantly express β2‐AR, with additional α1‐, α2‐, and β1‐subtypes [15, 16]. Hepatic stellate cells (HSCs) express α1‐ and β2‐AR, with α1‐AR being the principal mediator of fibrogenic responses [17, 18]. Kupffer cells, liver sinusoidal endothelial cells (LSECs), and cholangiocytes express various adrenergic receptor subtypes, with β2‐AR on Kupffer cells functionally linked to cytokine regulation and α1‐AR on LSECs and cholangiocytes contributing to vascular and biliary tone [14, 19, 20, 21].

#### 2.1.2 Parasympathetic Innervation

Parasympathetic regulation of the liver is mediated predominantly by the vagus nerve [14]. Preganglionic neurons in the dorsal motor nucleus of the vagus (DMV)—integrating inputs from hypothalamic nuclei including the PVN, VMH, and LHA—project to the liver via vagal hepatic branches [13, 22] (Figure 1C).

A distinctive feature is the absence of classical intrahepatic postganglionic ganglia. Current models propose that vagal preganglionic fibers may synapse at micro‐ganglia located near the celiac artery [23]. Although parasympathetic input to the hepatic parenchyma is generally limited [24], recent mouse studies identified choline acetyltransferase‐positive neurons within the liver, suggesting potential for local modulation [25]. In humans, cholinergic fibers are primarily localized along vasculature, with minimal hepatocyte contact [24] (Figure 1C).

The principal neurotransmitter is acetylcholine (ACh), which acts on muscarinic (mAChRs) and nicotinic receptors (nAChRs) expressed by liver cells (Figure 1D). Hepatocytes primarily express muscarinic receptor subtype 3 (M3R) and nAChR subunits Chrna2/4 [26, 27]. HSCs express multiple mAChRs and nAChR subunits, among which M3R and α7nAChR have been linked to fibrogenic activation [28, 29]. Kupffer cells and CD8^+^ T cells primarily express α7nAChR, which mediates the anti‐inflammatory effects for cholinergic signaling [30], and cholangiocytes express M1R, M3R, and α7nAChR [31, 32].

#### 2.1.3 Species‐Specific Variations and Compensatory Mechanisms

Hepatic autonomic innervation exhibits profound interspecies diversity [14]. Sympathetic and parasympathetic fibers consistently innervate the portal triad across mammals, but their density, distribution, and degree of parenchymal penetration vary substantially, with the most conspicuous difference in sympathetic innervation. Human and guinea pig livers show extensive intralobular TH‐positive sympathetic fibers contacting hepatocytes, whereas rodents show minimal parenchymal sympathetic innervation [14, 24]. By contrast, parasympathetic innervation is more conserved, with cholinergic fibers largely restricted to portal triads across species [24].

Compensatory mechanisms likely explain robust neurogenic regulation in species with sparse direct inputs, including electrotonic coupling via gap junctions between hepatocytes and indirect signaling via non‐parenchymal cells releasing secondary mediators (e.g., eicosanoids, nitric oxide) [33, 34]. Thus, autonomic control operates through three mechanisms: direct neurotransmission, gap junction propagation, and paracrine communication.

### 2.2 Hypothalamic‐Pituitary Axes in Hepatic Regulation

**FIGURE 2:**
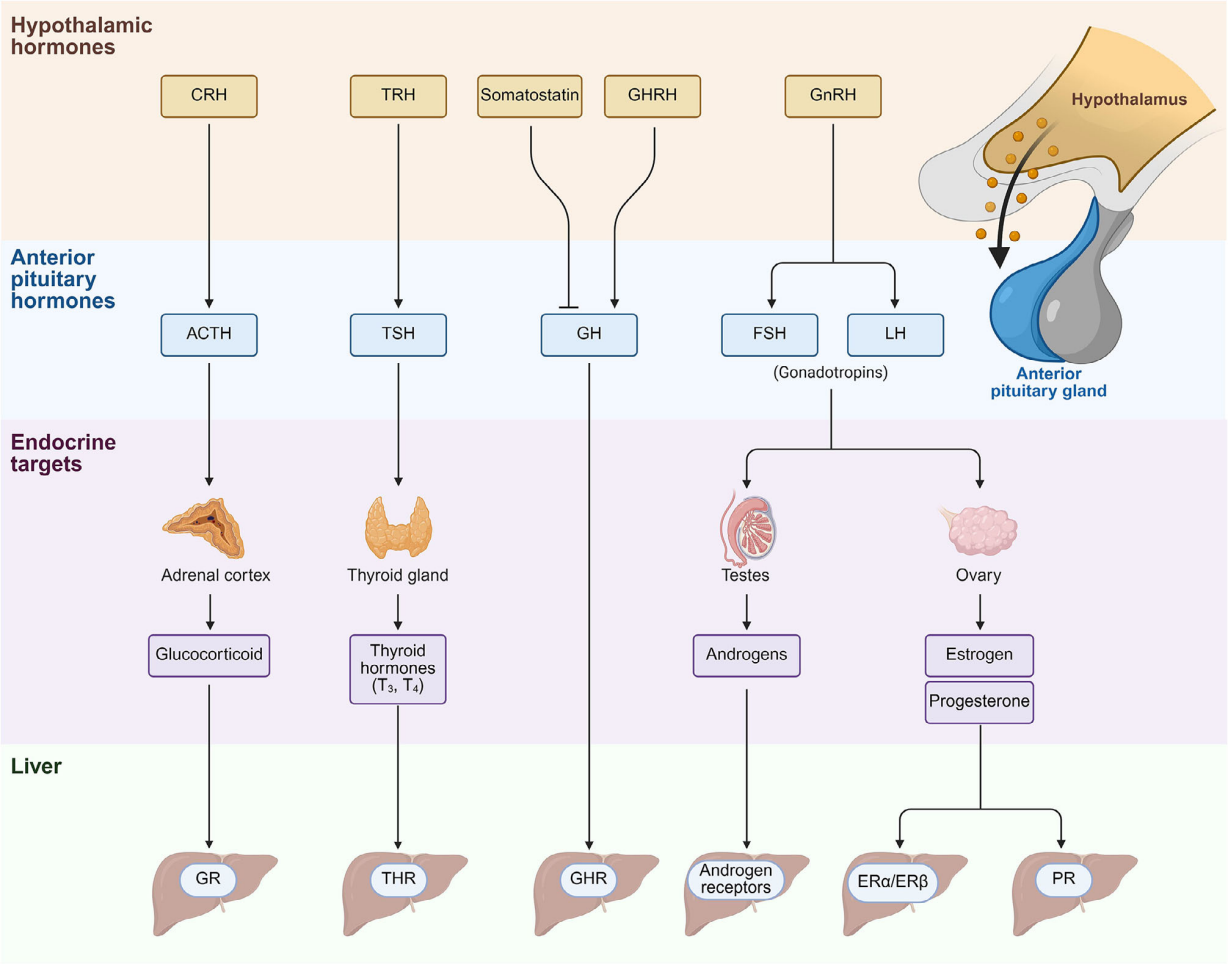
Hypothalamic‐pituitary axes in hepatic regulation. Schematic illustration of hypothalamic‐pituitary‐adrenal (HPA), hypothalamic‐pituitary‐thyroid (HPT), hypothalamic‐pituitary‐gonadal (HPG), and growth hormone (GH) axes, showing central inputs and hepatic receptor systems. ACTH, adrenocorticotropic hormone; CRH, corticotropin‐releasing hormone; ER, estrogen receptors; FSH, follicle‐stimulating hormone; GHR, GH receptor; GHRH, GH‐releasing hormone; GnRH, gonadotropin‐releasing hormone; GR, glucocorticoid receptors; LH, luteinizing hormone; PR, progesterone receptors; THR, thyroid hormone receptors; TRH, thyrotropin‐releasing hormone; TSH, thyroid‐stimulating hormone. Created in BioRender. Tang, Q. (2026) https://BioRender.com/bnfaatr.

The hypothalamus also governs hepatic homeostasis through classical neuroendocrine axes, including the hypothalamic‐pituitary‐adrenal (HPA), hypothalamic‐pituitary‐thyroid (HPT), hypothalamic‐pituitary‐gonadal (HPG), and growth hormone (GH) axes (Figure 2).

The HPA axis originates in corticotropin‐releasing hormone (CRH) neurons of the PVN, stimulating pituitary adrenocorticotropic hormone (ACTH) secretion and adrenal glucocorticoid production [35]. Hepatic glucocorticoid receptors (GR) mediate broad transcriptional regulation [36]. PVN CRH neurons receive input from AgRP neurons via melanocortin receptors (MC3R and MC4R), linking energy status to hepatic adaptation [37].

The HPT axis is governed by PVN thyrotropin‐releasing hormone (TRH) neurons, controlling pituitary thyroid‐stimulating hormone (TSH) release and circulating thyroid hormones (T3 and T4) [38]. Hepatic thyroid hormone receptors (THR) modulate lipid metabolism, inflammation, and fibrogenesis [38]. This axis is modulated by melanocortin signaling via MC4R on PVN TRH neurons [39].

The HPG axis involves gonadotropin‐releasing hormone neurons stimulating follicle‐stimulating hormone (FSH) and luteinizing hormone (LH), which regulate sex hormones that act on the liver [40, 41]. The liver expresses androgen, estrogen, and progesterone receptors, contributing to sexual dimorphism in hepatic metabolism [41, 42].

The GH axis exemplifies this integrative paradigm. The balance between hypothalamic GH‐releasing hormone and somatostatin neurons determines pulsatile GH secretion [43]. This pattern, through GH receptor engagement, establishes sexually dimorphic hepatic gene expression [43, 44].

## 3 Physiological Orchestration of Liver Function by the Hypothalamus–Liver Axis

The liver is the body's most metabolically flexible organ, dynamically reprogramming its pathways in response to nutrient availability and circadian signals [7, 45]. This section discusses the hypothalamus‐liver axis in regulating liver physiology, including glucose and lipid homeostasis, bile acid metabolism, and regenerative capacity, across fasting, feeding, and circadian cycles.

### 3.1 Hypothalamic Regulation of Hepatic Glucose Metabolism

The liver maintains glucose homeostasis by switching between glucose storage and production [45]. During fasting, it drives glucose output via glycogenolysis and gluconeogenesis, whereas feeding suppresses production and promotes storage [45]. Early models proposed opposing autonomic control, in which sympathetic activation stimulates glucose output during energy deficit and parasympathetic tone promotes storage after feeding [46]. However, emerging evidence reveals a more complex regulatory circuit.

#### 3.1.1 Hypothalamic Regulation of Hepatic Glucose Metabolism During Fasting

**FIGURE 3:**
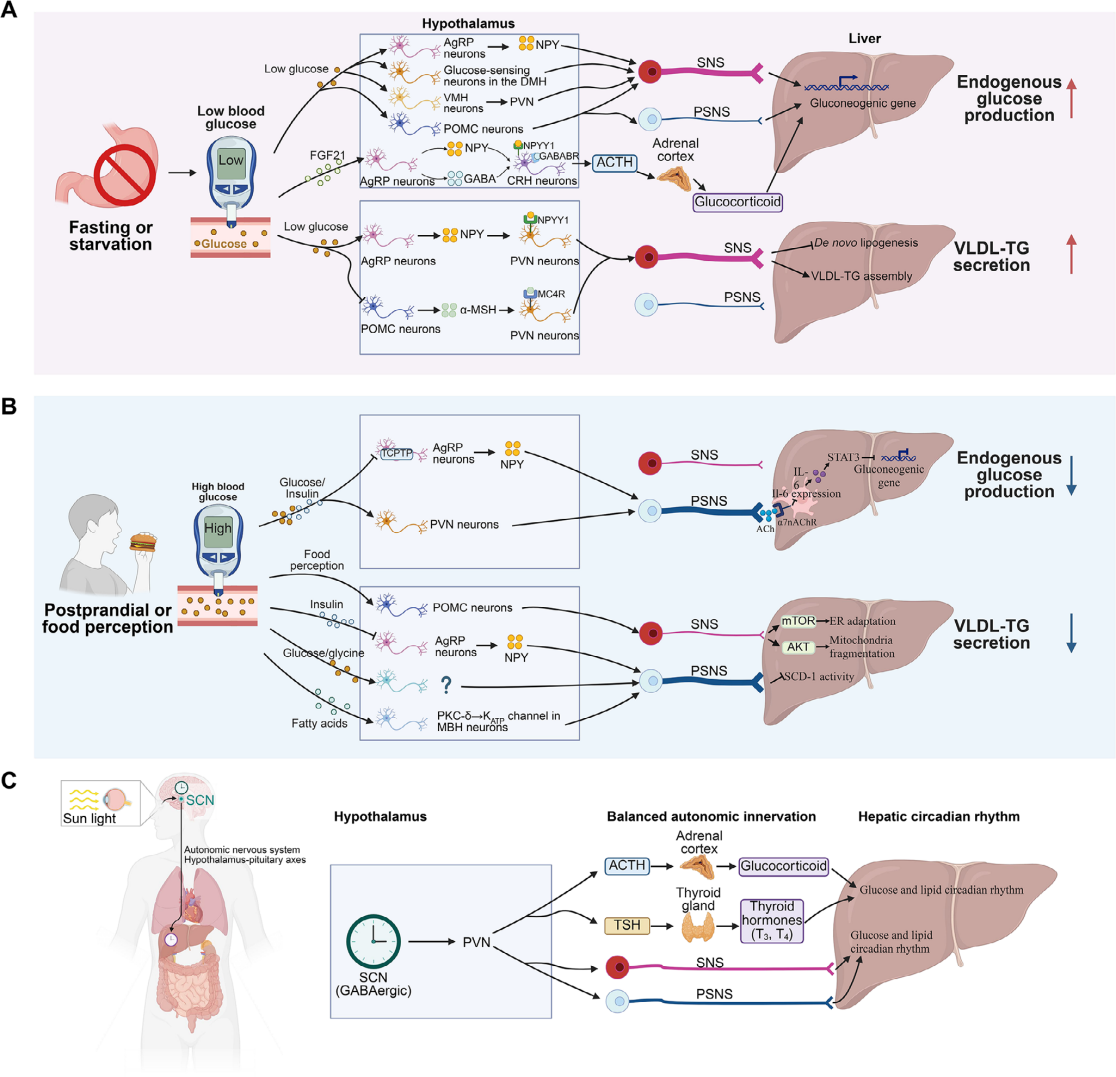
Physiological orchestration of hepatic glucose and lipid metabolism by the hypothalamus. (A) Hypothalamic regulation of hepatic glucose and lipid metabolism under fasting state. (B) Hypothalamic regulation under postprandial state or food perception. (C) Circadian regulation of hepatic glucose and lipid rhythm by the suprachiasmatic nucleus (SCN). α‐MSH, α‐melanocyte‐stimulating hormone; ACTH, adrenocorticotropic hormone; AgRP, agouti‐related protein; AKT, protein kinase B; CRH, corticotropin‐releasing hormone; DMH, dorsomedial hypothalamus; GABA, γ‐aminobutyric acid; GABABR, GABA‐B receptor; IL‐6, interleukin‐6; MC4R, melanocortin 4 receptor; mTOR, mammalian target of rapamycin; NPY, neuropeptide Y; NPYY1, NPY Y1 receptor; POMC, pro‐opiomelanocortin; PVN, paraventricular nucleus; SNS, sympathetic nervous system; PSNS, parasympathetic nervous system; SCD‐1, stearoyl‐CoA desaturase‐1; STAT3, signal transducer and activator of transcription 3; TSH, thyroid‐stimulating hormone; VLDL‐TG, very low‐density lipoprotein‐triglycerides; VMH, ventromedial hypothalamus. Created in BioRender. Tang, Q. (2026) https://BioRender.com/bnfaatr.

During fasting, the hypothalamus prevents hypoglycemia primarily via sympathetic outflow to the liver under the control of specific nuclei and neurons. AgRP neurons are rapidly activated by declining circulating glucose [37, 47]. NPY release from AgRP neurons directly counteracts hypoglycemia, and central NPY administration is sufficient to enhance endogenous glucose production [37, 47]. ARC POMC neurons also participate. Hypothalamic *Pomc* deletion impairs counterregulatory responses to hypoglycemia via the sympathetic pathways [48]. Interestingly, a recent study identified ARC POMC neurons projecting to the liver via the parasympathetic DMV [49]. Optogenetic stimulation of this pathway upregulates hepatic gluconeogenic enzymes and is sufficient to elevate blood glucose [49] (Figure 3A).

Other hypothalamic regions further fine‐tune this response (Figure 3A). Glucose‐sensing neurons in the DMH detect declining glucose levels and drive hepatic output through adrenergic signaling [50]. A study shows that prolonged fasting engages a multi‐synaptic circuit originating in the VMH, projecting to the PVN and extending to the lateral paragigantocellular nucleus. This pathway activates intrahepatic sympathetic nerves to stimulate glucose production. Within this circuit, *Galnt2* acts as a molecular brake on VMH glucose‐inhibited neurons, modulating the glycemic threshold for hypoglycemia detection [51].

Beyond direct autonomic control, the hypothalamus regulates fasting‐induced hepatic glucose production via the HPA axis (Figure 3A). HPA activation and subsequent glucocorticoid release mobilize fuel stores to prevent hypoglycemia [52]. During prolonged fasting, hepatic peroxisome proliferator‐activated receptor α‐induced fibroblast growth factor 21 (FGF21) crosses the blood‐brain barrier (BBB) to stimulate the HPA axis, establishing an adaptive mechanism for sustained energy deprivation [53]. Central NPY stimulates PVN CRH neurons via NPY Y1 receptor [37], while γ‐aminobutyric acid (GABA) released from AgRP neurons acts through GABA‐B receptors on PVN GABAergic afferents to CRH neurons, forming a disinhibitory circuit that amplifies glucocorticoid‐dependent glucose production [52].

#### 3.1.2 Hypothalamic Regulation of Hepatic Glucose Metabolism in the Postprandial State

Postprandially, insulin orchestrates a rapid transition from glucose production to utilization and storage. Beyond its direct action on the liver, insulin also acts centrally via receptors expressed in hypothalamic neurons to modulate hepatic glucose metabolism [54] (Figure 3B). Third‐ventricle infusion of insulin suppresses hepatic glucose production independent of peripheral insulin levels, whereas central antagonism of insulin signaling abolishes this effect [54].

AgRP neurons constitute a key cellular mediator of this central glucoregulatory action [55]. Selective ablation or restoration of insulin receptor in these neurons correspondingly impairs or rescues insulin‐mediated suppression of hepatic glucose production [55]. Central NPY administration disrupts insulin's effect [56]. In contrast, insulin receptor manipulation in POMC neurons affects energy expenditure without altering hepatic glucose metabolism [55]. Beyond the mediobasal hypothalamus, insulin‐sensitive PVN neurons also contribute by activating hepatic parasympathetic output to suppress glucose production [22]. Mechanistically, central insulin suppresses hepatic vagus activity, alleviating cholinergic inhibition of Kupffer cells and promoting hepatic interleukin‐6 (IL‐6)‐signal transducer and activator of transcription 3 (STAT3) signaling to downregulate gluconeogenic gene expression via α7nAChR [57].

#### 3.1.3 Circadian Regulation of Hepatic Glucose Metabolism via the SCN

The hypothalamus‐liver axis exhibits pronounced circadian rhythmicity, aligning hepatic glucose metabolism with the daily sleep‐wake cycle through autonomic and neuroendocrine pathways (Figure 3C). The central clock in the hypothalamic SCN synchronizes 24‐h rhythms in glucose metabolism [58]. SCN neurons are predominantly GABAergic and show firing rhythms that peak during the active phase [59]. This rhythm is disrupted following SCN lesioning, which also abolishes the cyclic expression of hepatic gluconeogenic genes [60]. SCN outputs to pre‐autonomic neurons in the PVN via GABAergic and glutamatergic projections establish autonomic balance for circadian glucose control [61]. Hepatic sympathectomy, but not parasympathectomy, prevents PVN GABA receptor blockade‐induced hyperglycemia [62]. Notably, while selective hepatic denervation of either branch disrupts glucose rhythms, complete denervation paradoxically preserves them, indicating that autonomic imbalance, not loss of innervation per se, impairs rhythmic regulation [63].

Neuroendocrine pathways complement neural regulation. SCN input to the PVN drives diurnal CRH release, resulting in peak glucocorticoid secretion at waking [64]. This HPA axis rhythm is essential for coordinating daily glucose homeostasis, as glucocorticoids promote hepatic gluconeogenesis [65]. Although inhibiting glucocorticoid synthesis does not abolish the morning rise [66], the HPA axis remains a critical component of the circadian glucose regulatory network. The HPT axis may also contribute given diurnal thyroid hormone variation [67], but direct evidence linking it to circadian hepatic glucose production remains limited.

Thus, the SCN provides circadian alignment for glucose rhythms, complementing the nutrient‐driven autonomic responses. Human evidence for these mechanisms remains limited.

### 3.2 Hypothalamic Regulation of Hepatic Lipid Metabolism

The liver balances triglycerides (TG) storage, mobilization, and export according to nutritional state. Fasting promotes very low‐density lipoprotein‐TG (VLDL‐TG) secretion through sympathetic activation [68], whereas postprandial parasympathetic activity suppresses this output [69]. This sympathetic‐parasympathetic opposition, initially established for glucose regulation, also applies to hepatic lipid handling [69], though recent findings point to additional circuit complexity.

#### 3.2.1 Hypothalamic Control of Hepatic Lipid Metabolism During Fasting

During prolonged fasting, the ARC serves as a key nutrient‐sensing hub that mediates metabolic adaptation via NPY signaling [68]. Fasting stimulates NPY release at sympathetic pre‐autonomic neurons in the PVN, which promotes sustained VLDL‐TG secretion. Central NPY administration rapidly increases hepatic VLDL‐TG output, whereas NPY receptor antagonists reduce elevated fasting VLDL output [70]. Mechanistically, NPY promotes VLDL‐TG assembly while suppressing *de novo* lipogenesis [70]. The sympathetic nervous system is the principal efferent pathway, as hepatic sympathectomy abolishes both fasting‐ and NPY‐induced VLDL‐TG secretion, whereas parasympathetic denervation has no effect [68]. In parallel, fasting suppresses POMC neuronal activity, reducing melanocortin signaling at MC4R‐expressing PVN neurons [71, 72]. This reciprocal regulation—NPY upregulation coupled with POMC downregulation—shifts the autonomic balance toward sympathetic dominance, reinforcing VLDL‐TG secretion during fasting [68] (Figure 3A).

#### 3.2.2 Hypothalamic Control of Postprandial Hepatic Lipid Metabolism

Following nutrient intake, the hypothalamus–liver axis orchestrates a coordinated transition from lipid mobilization to storage (Figure 3B). Hyperinsulinemia suppresses VLDL‐TG secretion, an effect abolished by central NPY administration, indicating that insulin inhibits AgRP neuronal activity to curtail hepatic lipid output in the fed state [56]. Central glucose infusion is sufficient to reduce VLDL‐TG secretion via a vagal pathway involving decreased hepatic stearoyl‐CoA desaturase‐1 (SCD‐1) activity [69, 73]. Beyond glucose, the hypothalamus detects circulating fatty acids via a protein kinase C‐δ (PKC‐δ)/K_ATP_ channel neurocircuit that signals through the dorsal vagal complex (DVC) to suppress VLDL‐TG secretion [74]. Central amino acid glycine administration similarly reduces VLDL‐TG secretion by activating DVC N‐methyl‐D‐aspartate (NMDA) receptors and inhibiting hepatic SCD‐1 [75].

The parasympathetic nervous system is a key conduit for postprandial inhibitory signals. Selective hepatic parasympathetic denervation elevates postprandial VLDL‐TG production, whereas sympathetic denervation also increases postprandial TG [69], indicating that sympathetic drive is suppressed after feeding. Thus, feeding simultaneously activates parasympathetic inhibition and withdraws sympathetic stimulation of VLDL‐TG secretion [69].

Metabolic preparation for nutrient intake begins even before ingestion. Sensory food cues activate hypothalamic POMC neurons, which stimulate sympathetic nerves projecting to the liver [76]. This induces hepatic mammalian target of rapamycin (mTOR) signaling, *Xbp1* splicing, endoplasmic reticulum (ER) stress genes, and phosphatidylcholine synthesis, indicating a melanocortin‐sympathetic‐mTOR‐XBP1s axis [76]. Food perception also triggers rapid hepatic mitochondrial fragmentation via protein kinase B (AKT)‐dependent phosphorylation of mitochondrial fission factor [77]. This rapid mitochondrial fragmentation is proposed to represent a preparative metabolic response that enhances hepatic nutrient processing efficiency in anticipation of incoming substrates, although its essentiality for postprandial adaptation and its relevance to metabolic disease remain to be determined.

#### 3.2.3 Circadian Regulation of Hepatic Lipid Metabolism via the SCN

Hepatic lipid metabolism is subject to circadian regulation, in which the SCN plays a central role [78] (Figure 3C). The SCN drives circadian glucocorticoid secretion via the HPA axis. Chronic activation of the HPA axis alone alters circadian expression of hepatic clock and lipid metabolism‐related genes without affecting clock gene expression in the SCN itself [79]. The HPT axis is also regulated by the SCN. SCN lesioning abolishes circadian oscillations in circulating TSH and thyroid hormones [80].

In addition, mice exposed to artificial light at night show upregulation of hepatic lipogenic genes [81]. This light‐induced signal is transmitted from the SCN to the liver via the autonomic nervous system, as selective hepatic denervation abolishes the effects of nocturnal light on hepatic gene expression [82]. However, whether these circadian regulatory mechanisms operate similarly in human physiology remains to be established.

### 3.3 Hypothalamic Regulation of Hepatic Biliary Metabolism

Autonomic innervation also modulates bile metabolism. Specifically, cholangiocytes actively participate in bidirectional neuro‐biliary crosstalk [83]; they express receptors for multiple neurotransmitters and can synthesize neurotransmitters *de novo*. Consistently, efferent hepatic innervation helps regulate bile flow [84].

Postprandial parasympathetic activation serves as a major stimulus for biliary bicarbonate secretion, a process that likely operates during the digestive phase when parasympathetic tone is elevated and secretin acts on the biliary epithelium [85]. ACh enhances secretin‐induced bicarbonate secretion via M3R, which is abundantly expressed on cholangiocytes [86]. Vagotomy reduces cholangiocyte M3R expression and blunts secretin‐stimulated bile flow [87].

Sympathetic signaling also fine‐tunes cholangiocyte function. TH‐positive adrenergic nerve fibers are closely associated with bile ducts [83]. Cholangiocytes uniformly express α2‐AR, and activation of these receptors counterbalances cholinergic stimulation by reducing intracellular cAMP levels, thereby attenuating secretin‐induced choleresis [88].

The HPA axis represents a distinct neuroendocrine pathway linking the hypothalamus to bile metabolism. Glucocorticoids influence the expression of bile acid transporters, including the bile salt export pump and sodium‐taurocholate co‐transporting polypeptide, thereby modulating bile flow and composition [89]. The farnesoid X receptor and the G protein‐coupled receptor TGR5, activated by bile acids, are expressed in both the liver and the hypothalamus, potentially serving as molecular links for neuroendocrine feedback [90].

Thus, hepatic biliary metabolism is controlled by parasympathetic and sympathetic neural inputs, with the HPA axis and bile acid receptors constituting further regulatory layers. However, human evidence for this regulation remains unclear.

### 3.4 Hypothalamic Regulation of Liver Regeneration

The liver possesses extraordinary regenerative capacity following partial hepatectomy or injury, a process under sophisticated neural control via the hypothalamus–liver axis. Both sympathetic and parasympathetic branches contribute, though their roles exhibit distinct patterns.

#### 3.4.1 Parasympathetic Promotion of Regeneration

The parasympathetic nervous system is a key driver of liver regeneration. Hepatic vagotomy is sufficient to substantially impair the regenerative response after partial hepatectomy [91]. Enhancing parasympathetic outflow through VMH lesions markedly drives regeneration, an effect abolished by simultaneous vagotomy [92].

**FIGURE 4:**
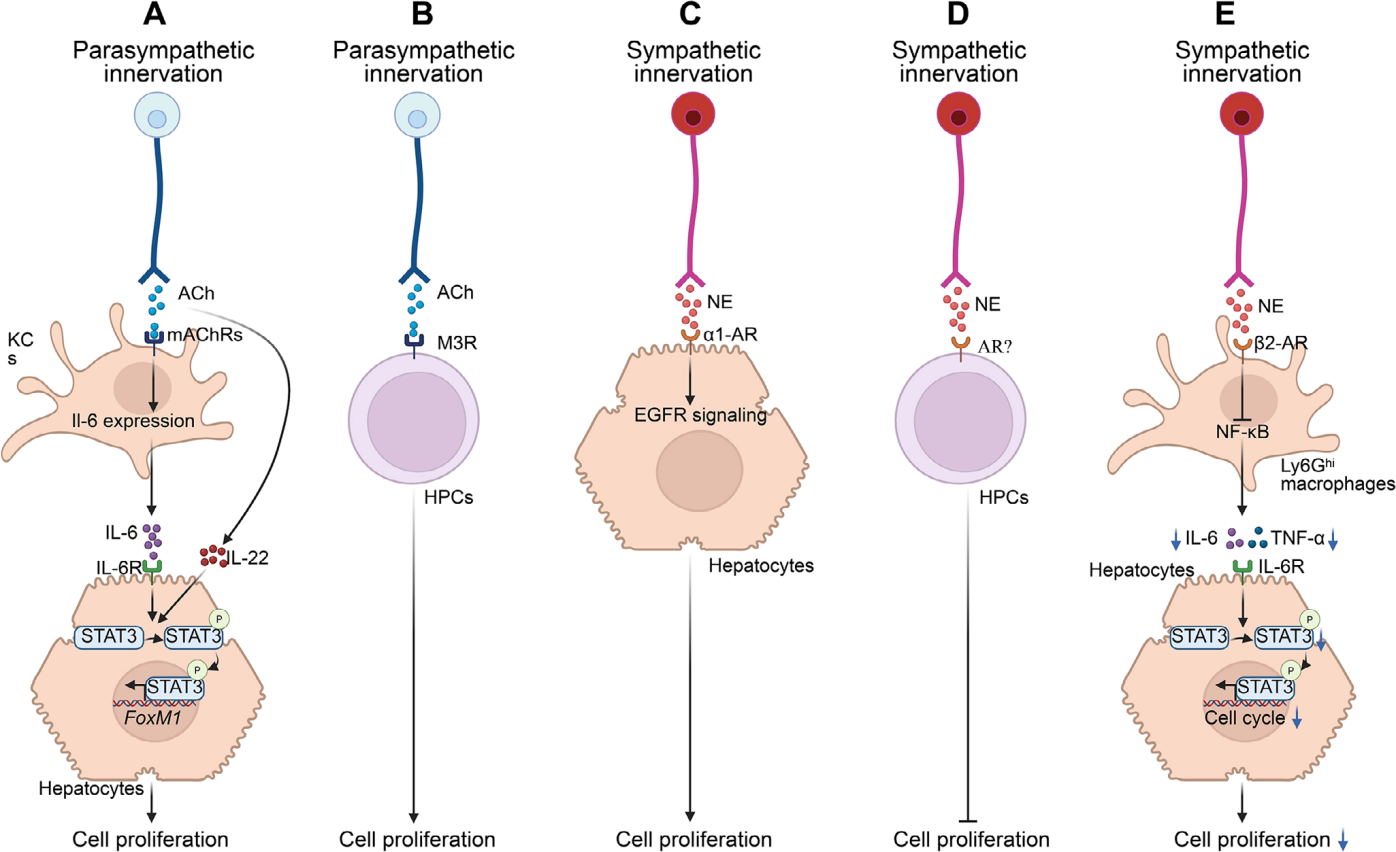
Neural regulation of liver regeneration. Parasympathetic (A, B) and sympathetic (C–E) pathways controlling liver cell proliferation via ACh and NE signaling, respectively. Pathways involve Kupffer cell (KC)/macrophage and hepatocyte crosstalk (A, E), direct hepatic progenitor cell (HPC) regulation (B, D), and direct hepatocyte activation (C). ACh, acetylcholine; AR, adrenergic receptors; IL‐6, interleukin‐6; IL‐6R, interleukin‐6 receptor; M3R, muscarinic M3 receptor; NE, norepinephrine; NF‐κB, nuclear factor κ‐B; STAT3, signal transducer and activator of transcription 3; TNF‐α, tumor necrosis factor α. Created in BioRender. Tang, Q. (2026) https://BioRender.com/bnfaatr.

Mechanistically, ACh released from vagal terminals activates Kupffer cells to produce IL‐6, which then activates STAT3 and FoxM1 signaling in hepatocytes, driving DNA synthesis and cellular proliferation [93]. Hepatic branch vagotomy suppresses FoxM1 upregulation and impairs hepatocyte proliferation, whereas restoring FoxM1 reverses these deficits [93]. Emerging evidence also implicates IL‐22 as an additional parasympathetic effector downstream of vagal signaling [94]. Vagotomy reduces hepatic IL‐22 production after partial hepatectomy, and IL‐22 administration promotes proliferation via STAT3 activation, partially rescuing the regenerative defect [94] (Figure 4A).

Beyond mature hepatocytes, parasympathetic signaling also modulates hepatic progenitor cells (HPCs). In human livers, HPCs express the M3R [95]. Cholinergic signaling directly regulates HPC dynamics via this receptor, as vagotomy reduces HPC numbers in rats with drug‐induced hepatitis, and transplanted (denervated) human livers show fewer HPCs during hepatitis compared with native livers [95] (Figure 4B).

#### 3.4.2 Context‐Dependent Sympathetic Regulation of Liver Regeneration

Sympathetic regulation of liver regeneration is complex and context‐dependent. NE released from sympathetic terminals enhances hepatocyte proliferation by potentiating epidermal growth factor (EGF) signaling via α1‐AR [96] (Figure 4C). The α1‐AR antagonist prazosin blocks both EGF receptor downregulation and the associated increase in DNA synthesis [96].

Conversely, evidence also points to an inhibitory role of sympathetic activity. Inhibiting sympathetic nerve activity expands HPC populations [97] (Figure 4D). Recent work shows that sympathetic nerves restrain regeneration via NE acting on Kupffer cell β2‐AR, which suppresses nuclear factor κB (NF‐κB) activation, reduces IL‐6 and tumor necrosis factor α (TNF‐α) production, and subsequently impairs hepatocyte STAT3 signaling and proliferation [98] (Figure 4E). This paradox underscores the complexity of sympathetic regulation, with effects depending on AR subtypes, macrophage activation state, and regenerative context. Whether this complexity applies to human liver regeneration remains to be determined.

## 4 Hypothalamus–Liver Axis Dysregulation in Liver Diseases

The hypothalamus–liver axis maintains metabolic homeostasis under physiological conditions. However, chronic nutrient excess or pathological stress can render this axis maladaptive, driving the onset of liver diseases. Here we examine how dysregulation of this axis disrupts glucose metabolism and promotes diabetes, fuels lipid accumulation and inflammation in MASLD, ALD/MetALD. We then address the broader landscape of liver pathology—including injury, fibrosis, cirrhosis, and HCC—in which hypothalamus‐liver axis dysregulation has been implicated, and conclude with the consequence of hypothalamic‐pituitary disorders and their surgical treatments in liver disease.

### 4.1 Dysregulation in Glucose Metabolism

Autonomic dysregulation is a hallmark of diabetes. Experimental models reveal hepatic autonomic neuropathy, characterized by reduced NE overflow and blunted glucose output [99]. In parallel, hypothalamic insulin signaling is also compromised in diabetic rats [100]. These peripheral and central defects are accompanied by chronically elevated NPY levels, which sustain hyperglycemia in obesity [56, 101]. Mechanistically, central NPY administration impairs insulin‐mediated suppression of hepatic glucose production via a sympathetic pathway [56, 101].

These functional alterations correspond with electrophysiological remodeling in key hypothalamic nuclei. For instance, liver‐projecting PVN neurons in diabetic *db/db* mice exhibit heightened spontaneous activity [102]. High‐fat feeding similarly increases PVN neuronal excitability and induces insulin resistance at the single‐cell level [103]. Direct PVN stimulation with NMDA or GABA‐A receptor blockade evokes sympathetically mediated hyperglycemia [104], an effect potentiated under high‐fat diet conditions via hypothalamic apelin/reactive oxygen species (ROS) signaling [105]. Thus, PVN hyperexcitability serves as a neural basis and driver for the sympathetic overdrive and glucose dysregulation observed in diabetes.

ARC POMC neurons also regulate hepatic glucose metabolism. Their chemogenetic activation reduces hepatic glucose output [106], and activation by leptin and glucagon‐like peptide (GLP)‐2 improves insulin sensitivity and glucose homeostasis [107, 108]. Transgenic POMC expression in leptin‐deficient obese mice normalizes hyperglycemia [109].

Classical neuroendocrine axes also contribute to diabetic pathogenesis. Clinical observations, such as the association between lower FSH levels and increased diabetes risk in postmenopausal women, implicate neuroendocrine disruption in diabetes [110]. Experimental disruption of GH signaling in specific hypothalamic neurons impairs insulin‐mediated hepatic glucose regulation independently of adiposity or circulating hormones [111]. Thus, neuroendocrine signals operate in parallel with autonomic pathways to modulate glucose homeostasis.

Beyond homeostatic pathways, stress‐responsive circuits are potent drivers of glucose dysregulation. Acute stress engages a polysynaptic circuit from the medial amygdala to the liver via VMH neurons and hepatic sympathetic nerves, rapidly stimulating hepatic gluconeogenesis independently of canonical neuroendocrine stress axes [112]. Chronic stress remodels this circuitry, leading to persistent, diabetes‐like glucose dysregulation [112]. Whether stress‐induced remodeling of this circuitry contributes to human diabetes remains to be determined.

### 4.2 Dysregulation in MASLD Pathogenesis

**FIGURE 5:**
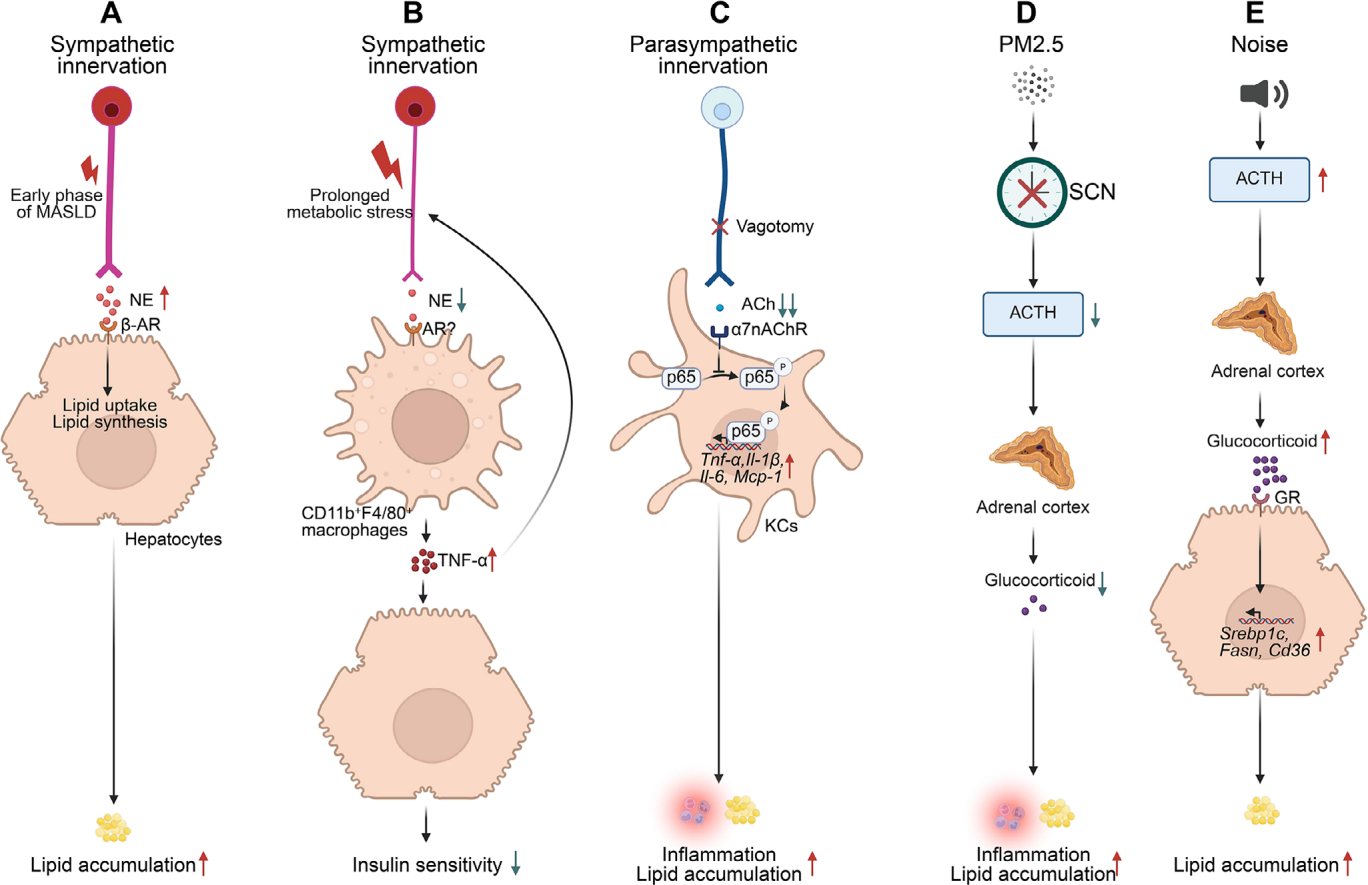
Hypothalamic‐liver axis dysregulation in metabolic dysfunction‐associated steatotic liver disease (MASLD). (A) Sympathetic hyperactivation in early MASLD: NE/β‐AR signaling upregulates hepatic lipid uptake and synthesis genes, promoting steatosis. (B) Prolonged metabolic stress triggers sympathetic neurodegeneration via TNF‐α‐Sarm1 signaling, depleting NE, exacerbating inflammation, and decreasing insulin sensitivity. (C) Loss of parasympathetic protection: reduced ACh/α7nAChR signaling relieves NF‐κB suppression, increasing pro‐inflammatory cytokines and lipid accumulation. (D) PM2.5 disrupts SCN and adrenal circadian rhythms, suppressing HPA axis and glucocorticoid synthesis, promoting inflammation and steatosis. (E) Chronic noise activates HPA axis, elevating glucocorticoids and GR target genes, inducing MASLD. ACh, acetylcholine; ACTH, adrenocorticotropic hormone; AR, adrenergic receptors; GR, glucocorticoid receptors; KCs, Kupffer cells; NE, norepinephrine; SCN, suprachiasmatic nucleus; TNF‐α, tumor necrosis factor α. Created in BioRender. Tang, Q. (2026) https://BioRender.com/bnfaatr.

MASLD, previously termed nonalcoholic fatty liver disease (NAFLD), is defined as hepatic steatosis in the presence of at least one cardiometabolic risk factor and the absence of other specific etiologies [113]. Autonomic imbalance is a hallmark of MASLD. The autonomic nervous system exhibits a dynamic, biphasic role in disease progression. Disease onset is associated with hepatic sympathetic hyperactivation, with experimental models showing an approximate doubling in sympathetic nerve firing rates [114]. This heightened tone directly promotes steatosis, as surgical hepatic sympathectomy mitigates diet‐induced lipid deposition without altering energy balance [114]. Mechanistically, sympathetic signaling upregulates hepatic genes involved in lipid uptake and synthesis [114] (Figure 5A). Conversely, prolonged metabolic insult triggers sympathetic nerve pathology. Advanced imaging in human MASLD and animal models reveals initial axonal sprouting followed by progressive neurodegeneration in steatohepatitis [24, 115], a degenerative process mediated partially via macrophage‐dependent TNF‐α‐Sarm1 signaling that depletes local NE and exacerbates inflammation and metabolic dysfunction [24] (Figure 5B). In contrast, vagal parasympathetic signaling generally exerts protective, anti‐inflammatory effects [116]. Vagotomy exacerbates liver inflammation by disrupting α7nAChR on Kupffer cells, a pathway that suppresses NF‐κB activity and reduces pro‐inflammatory cytokine production [117] (Figure 5C).

The hypothalamus integrates nutrient and hormonal signals into autonomic commands that regulate hepatic lipid metabolism. Under obesogenic conditions, PVN neurons show increased excitability and impaired insulin responsiveness, contributing to elevated sympathetic output [103]. Sustained central NPY elevation stimulates hepatic lipogenesis and hypertriglyceridemia [70], likely through tonic activation of PVN sympathetic pre‐autonomic neurons that drive excessive VLDL‐TG secretion [68, 70, 118]. In AgRP neurons, p53 protects against diet‐induced obesity by suppressing hypothalamic c‐Jun N‐terminal kinase (JNK) activity, and its ablation increases susceptibility to obesity and metabolic dysfunciton [119]. Conversely, central melanocortin receptor activation suppresses hepatic lipogenic programs [120].

Several circulating hormones and nutrients engage these central circuits to modulate hepatic lipid homeostasis. Central GLP‐1 and leptin signaling both reduce hepatic lipid content through dual autonomic branches [121, 122, 123]. Leptin's anti‐steatotic effects, which involve hepatic AMP‐activated protein kinase (AMPK) activation and SCD‐1 downregulation, originate in the DVC and require intact vagal innervation, a finding supported by human studies [122, 124]. Other central modulators further fine‐tune hepatic lipid handling: central T3 promotes lipogenesis via parasympathetic outflow dependent on AMPKα1 in VMH neurons, which in turn regulates JNK1 activation to control hepatic lipid metabolism [125]; whereas deletion of the regulatory subunit AMPKγ2 leads to hepatic lipid accumulation [126]. Orexin protects against steatohepatitis by attenuating hepatic ER stress through autonomic pathways [127]. Free acid receptor‐1 in POMC neurons functions as a nutrient sensor for circulating fatty acids; its deficiency promotes hepatic insulin resistance and steatosis [128].

Dysregulation extends to hypothalamic‐pituitary axes. Environmental stressors influence MASLD susceptibility through the HPA axis. Chronic PM2.5 exposure suppresses HPA axis activity by disrupting circadian rhythms of clock genes in the hypothalamus and adrenal gland, leading to reduced glucocorticoid synthesis, which promotes hepatic steatosis [129] (Figure 5D). Conversely, chronic noise exposure activates the HPA axis, elevating plasma ACTH and glucocorticoid levels. Excessive glucocorticoid acts directly on hepatic GR to increase target gene transcription, thereby inducing MASLD [130] (Figure 5E). Similarly, disruptions in the gonadal axis, diminished THR‐β signaling, GH deficiency, and reduced prolactin levels are all robustly associated with aggravated hepatic lipid accumulation [131, 132, 133]. Notably, hormone replacement therapies targeting these axes have shown therapeutic potential in preclinical models [134, 135]. Emerging evidence also indicates that FSH regulates hepatic lipid metabolism via pituitary paracrine signaling or direct action on hepatocyte‐expressed receptors, independently of sex steroids [136, 137], whereas altered LH levels have been associated with MASLD risk in clinical studies [138, 139].

Sex differences in hypothalamic regulation of MASLD are increasingly recognized [140, 141]. The hypothalamus exhibits sexual dimorphism in response to metabolic signals, driven by organizational effects of neonatal androgens that irreversibly program hypothalamic centers controlling hepatic metabolism [142]. Estrogens exert protective effects on hepatic lipid metabolism, whereas androgens promote visceral and ectopic fat deposition [141]. In male mice, diet‐induced MASLD triggers hypothalamic inflammation, while females show intestinal remodeling. Over time, males develop more severe hepatic pathology, whereas females maintain metabolic preservation [143]. These sex‐specific responses highlight the need for sex‐specific therapeutic strategies, though their mechanistic basis remains largely unexplored.

### 4.3 Dysregulation in ALD and MetALD

ALD is driven by harmful alcohol use (>60 g/day in men or >50–60 g/day in women). MetALD describes patients meeting MASLD criteria with intermediate alcohol intake (210–420 g/week for men; 140–350 g/week for women), reflecting a dual burden of metabolic dysfunction and moderate alcohol consumption [144, 145]. While the hypothalamus–liver axis has been extensively studied in MASLD, its dysregulation in ALD and MetALD is less well characterized but emerging evidence points to overlapping yet distinct patterns.

Autonomic dysregulation is a hallmark of ALD and likely contributes to MetALD. Parasympathetic dysfunction is particularly common in ALD, affecting up to 72%–77% of patients in end‐stage liver disease, and is further exaggerated by alcohol consumption [146]. Enhanced central sympathetic outflow drives alcohol‐induced hepatic steatosis and metabolic dysregulation, mirroring MASLD but with distinct drivers [147, 148]. MetALD patients exhibit a metabolic phenotype closer to ALD, suggesting similar autonomic dysregulation, though direct studies are needed.

Hypothalamic insulin resistance directly contributes to ALD pathogenesis. In rodent models, acute alcohol impairs hypothalamic insulin action via increased hypothalamic inflammation and protein tyrosine phosphatase 1B upregulation [149]. Hypothalamic insulin signaling decreases after binge drinking, whereas hepatic signaling remains intact, indicating a primary central effect [149]. Subsequent studies have confirmed that this brain insulin resistance, via sympathetic overdrive and adipose tissue lipolysis, drives hepatic steatosis, a key initiating event in ALD pathogenesis [147].

Hypothalamic AgRP neurons also contribute to alcohol‐induced hepatic steatosis. Acute alcohol exposure increases AgRP neuron activity and immunoreactivity [150]. AgRP deficiency reduces hepatic lipid accumulation after binge‐like alcohol consumption, and the sympathetic nervous system is critical for alcohol‐induced steatosis [150]. Central adenosine A2B receptor signaling modulates alcohol‐induced fatty liver, and A2B‐deficient mice are resistant to steatosis [150]. These findings establish AgRP neurons and central adenosine signaling as crucial mediators of alcohol‐induced hepatic lipid accumulation. Additionally, hypothalamic β‐endorphin neuron transplantation into the PVN attenuates alcohol‐induced hepatic steatosis and fibrosis in rats, possibly via modulating autonomic outflow and hepatic immune function [151].

Notably, current evidence for hypothalamic dysregulation in ALD and MetALD is largely derived from rodent models, and whether these mechanisms operate in humans remains unclear.

### 4.4 Dysregulation in Hepatic Inflammation and Injury

The liver is highly susceptible to stressors, which trigger sequential inflammatory, reparative, and fibrotic responses. During the initial injury phase, the autonomic nervous system exerts dichotomous regulatory effects, as detailed in Table 1.

**TABLE 1:** Autonomic nervous system regulation of hepatic inflammation, injury, and fibrosis.

Pathological process	Therapeutic targets/molecules	Drug or intervention	Animal/cell model	Mechanism	Outcome
Sympathetic regulation					
D‐galactosamine‐induced acute liver injury [152]	α1‐AR	Nerve stimulation; NE; Phenylephrine (agonist)	Rats	Activated α1‐AR and increased Ca^2^^+^ influx	Increased LDH and AST release; exacerbated liver injury
HMCDE diet‐induced chronic liver injury [97]	α1‐AR	Prazosin (antagonist); 6‐OHDA	Mice	Blocked α1‐AR or depleted sympathetic nerves; increased oval cell accumulation	Reduced liver enzymes, necrosis, and steatosis
CCl4‐induced acute liver injury [153]	Sympathetic nerves	6‐OHDA	Rats	Depleted hepatic sympathetic nerves and improved energy metabolism	Alleviated liver injury but impaired liver regeneration
CCl4‐induced acute hepatotoxicity [154]	Sympathetic nerves	6‐OHDA	Mice	Reduced lipid peroxidation and pro‐inflammatory cytokines	Reduced liver enzymes, necrosis, and steatosis
CCl4‐induced acute and chronic liver injury [175]	Sympathetic nerves	6‐OHDA	WKY rats; HepG2 cells	Suppressed TGF‐β1‐induced hepatocyte apoptosis; increased hepatocyte proliferation	Decreased apoptosis and increased proliferation
Sinensis‐induced hepatobiliary injury [234]	β2‐AR	β2‐AR deficiency	Mice; Macrophages	Impaired M2 macrophage polarization	Alleviated hepatobiliary damage
LPS‐induced acute liver injury [156]	β2‐AR	Clenbuterol (agonist)	Rats	Activated β2‐AR; suppressed pro‐inflammatory cytokines	Reduced liver damage
Fas‐induced liver injury [155]	β2‐AR	Clenbuterol, Salbutamol (agonist)	Mice	Activated β2‐AR; inhibited Fas‐mediated hepatocyte apoptosis	Prevented liver apoptosis and death
Acetaminophen‐induced acute liver injury [235]	α1‐AR	Prazosin, doxazosin, terazosin, tamsulosin (antagonist)	Mice	Blocked α1‐AR; prevented hepatic erythrocyte accumulation and microcirculatory disturbance	Prevented hepatotoxicity
Acetaminophen‐induced acute liver injury [236]	β‐AR	Isoproterenol (agonist)	Mice	Activated and expanded HPCs via Wnt/β‐catenin pathway	Reduced liver injury and improved survival
MASH [157]	β‐AR	Propranolol (antagonist)	MASH mouse model	Blocked β‐AR on hepatocytes and induced hepatocyte apoptosis	Worsened liver injury
Liver fibrosis [167]	α1A‐AR	NE	Human HSCs	Activated α1A‐AR; induced calcium spikes and activated NF‐κB	Promoted HSC contraction and chemokine secretion
MASLD‐associated liver fibrosis [17]	α/β‐AR;NPY receptors	NE, Epi, NPY	Human HSCs; human MASLD liver	Activated α/β‐AR and NPY receptors; stimulated HSC proliferation and induced TGF‐β and collagen	Promoted HSC proliferation and fibrogenesis
Liver fibrosis [166]	α/β‐AR	NE	Mice; rats; murine HSCs	Activated AR via G protein/PI3K/MAPK and upregulated TGF‐β and collagen	Promoted HSC activation and hepatic fibrosis
Primary sclerosing cholangitis [168]	β‐AR	Propranolol (antagonist)	Mdr2^−/−^ mice	Blocked β‐AR; reduced periportal expression of TNF‐α, TGF‐β, CTGF, procollagen 1A1, angiotensinogen, and endothelin‐1	Reduced inflammation and periportal fibrosis; improved liver architecture
Parasympathetic regulation					
LPS‐induced inflammation [237]	α7nAChR	Nicotine (agonist), ACh, vagus nerve stimulation	Human macrophages; rats	Post‐transcriptional inhibition of cytokine synthesis	Reduced pro‐inflammatory cytokines; attenuated lethal endotoxic shock
Fas‐induced fulminant hepatitis [158]	α7nAChR	Nicotine, PNU‐282987 (agonist)	Mice with or without hepatic vagotomy	Reduced KCs ROS production	Reduced hepatocyte apoptosis and mortality
Hepatic ischemia‐reperfusion injury [159]	α7nAChR	PNU‐282987 (agonist)	Mice; KCs and hepatocytes	Reduced KCs ROS and H_2_O_2_ production; reduced hepatocyte apoptosis	Reduced liver injury
Hepatic ischemia‐reperfusion injury [162]	α7nAChR	PNU‐282987 (agonist)	Mice	Inhibited NF‐κB/HMGB1 and suppressed TNF‐α/IL‐1β	Reduced transaminase levels and hepatic inflammation
Hepatic ischemia‐reperfusion injury [163]	α7nAChR	Nicotine (agonist)	Mice	Activated PI3K/Akt/Nrf2; induced HO‐1; inhibited NF‐κB/HMGB1	Reduced oxidative stress, transaminase levels and hepatic injury
Acetaminophen‐induced liver injury [160]	Acetylcholinesterase (AChE)	Neostigmine (AChE inhibitor)	Mice	Inhibited AChE; activated Cholinergic anti‐inflammatory pathways; reduced TNF‐α and IL‐1β	Reduced liver enzymes and necrosis; improved survival
Acetaminophen‐induced liver injury [161]	Central mAChR and α7nAChR	Donepezil, rivastigmine, huperzine A (AChE inhibitor)	Mice	Reduced central ROS and JNK2 phosphorylation	Reduced liver enzymes and necrosis; protected mitochondrial function
Bacterial infection / Sepsis [238]	Cholinergic circuit	Vagus nerve stimulation	Mice	Enhanced KCs phagocytic capacity via nAChR and mAChR	Reduced hepatic bacterial load; promoted anti‐inflammatory phenotype
CCl4‐induced liver fibrosis [239]	Cholinergic fibers	—	Rats	Cholinergic nerve terminals formed close contacts with mast cells and myofibroblasts in fibrous septa	Formed mast cell/myofibroblast/nerve complexes and promoted fibrosis
Liver fibrosis [240]	nAChR	ACh	Murine HSCs	Activated nAChR on HSCs and induced collagen gene expression	Promoted HSC proliferation and collagen expression
MASH‐associated liver fibrosis [169]	M2/M3 mAChR	ACh	Primary human HSCs, human MASH liver	Activation of PI3K and MEK signaling; upregulation of M2/M3 receptors	Promoted HSC proliferation and fibrogenesis
CCl4‐induced liver fibrosis [170]	M1, M3, M5 mAChR	ACh, pilocarpine (agonist), atropine (antagonist)	Rat; primary rat hepatocytes	Activation of mAChR; elevation of hydroxyproline and collagen I/III	Promoted hepatocyte injury and hepatic fibrosis. Atropine ameliorated fibrotic changes
Hepatic fibrosis [241]	nAChR	ACh, mecamylamine (antagonist)	Cultured HSCs	Activation of nicotinic receptor	Increased HSC proliferation and collagen expression (blocked by mecamylamine)

**Abbreviations: **6‐OHDA, 6‐hydroxydopamine; ACh, acetylcholine; AKT, protein kinase B; AR, adrenergic receptor; AST, aspartate aminotransferase; CCl4, carbon tetrachloride; CTGF, connective tissue growth factor; Epi, epinephrine; HMCED, half methionine choline‐deficient diet plus ethionine; HMGB1, high mobility group box 1; HO‐1, heme oxygenase‐1; HPC, hepatic progenitor cell; HSC, hepatic stellate cell; IL‐1β, interleukin‐1β; JNK2, c‐Jun N‐terminal kinase 2; KCs, Kupffer cells; LDH, lactate dehydrogenase; LPS, lipopolysaccharide; mAChR, muscarinic acetylcholine receptor; MAPK, mitogen‐activated protein kinase; MASH, metabolic dysfunction‐associated steatohepatitis; MASLD, metabolic dysfunction‐associated steatotic liver disease; MEK, mitogen‐activated protein kinase; nAChR, nicotinic acetylcholine receptor; NE, norepinephrine; NF‐κB, nuclear factor κB; NPY, neuropeptide Y; Nrf2, nuclear factor erythroid 2‐related factor 2; PI3K, phosphoinositide 3‐kinase; ROS, reactive oxygen species; TGF‐β1, transforming growth factor β 1; TNF‐α, tumor necrosis factor α; α7nAChR, α7 Nicotinic acetylcholine receptor.

Sympathetic activation generally aggravates hepatic injury. Hepatic sympathetic nerve stimulation is sufficient to exacerbate acute liver injury via α1‐AR [152], whereas chemical sympathectomy reduces hepatocellular necrosis and pro‐inflammatory cytokines [97, 153, 154]. However, the role of sympathetic signaling is context‐dependent. For instance, β2‐AR activation can be protective under certain conditions [155, 156], whereas β‐antagonists such as propranolol have been reported to worsen liver injury in some mouse models of MASLD [157].

In contrast, the parasympathetic nervous system mediates anti‐inflammatory and protective functions. Vagal stimulation attenuates Fas‐induced hepatocyte apoptosis via α7nAChR on Kupffer cells, whereas vagotomy exacerbates liver injury [158]. In hepatic ischemia‐reperfusion injury, vagal activation reduces hepatic apoptosis by suppressing Kupffer cell‐derived ROS [159]. Therapeutically, acetylcholinesterase inhibitors and α7nAChR agonists confer protection in acetaminophen‐induced liver injury [160, 161, 162, 163]. However, these protective effects have been demonstrated primarily in acute injury models, and whether they translate to chronic liver inflammation or human disease remains uncertain.

### 4.5 Dysregulation in Hepatic Fibrosis

The transition from injury to fibrosis is coordinately regulated by autonomic pathways, as detailed in Table 1. The sympathetic nervous system establishes a direct neuro‐fibrogenic axis via HSCs [164]. Sympathetic overactivity exacerbates carbon tetrachloride‐induced fibrosis in mice, whereas reduced sympathetic tone confers protection [165]. NE promotes HSC proliferation and a pro‐fibrotic phenotype [17, 166, 167], and β‐adrenergic blockade with propranolol attenuates experimental sclerosing cholangitis [168].

The parasympathetic nervous system also exerts pro‐fibrogenic effects, primarily through mAChR‐mediated HSC activation. Exogenous ACh enhances HSC proliferation and fibrogenic marker expression via mAChRs [169]. The mAChR agonist pilocarpine elevates collagen levels, an effect reversed by atropine [170]. Thus, the autonomic regulation of fibrosis involves both sympathetic and parasympathetic pro‐fibrogenic pathways.

### 4.6 Dysregulation in Cirrhosis

Cirrhosis is associated with elevated circulating catecholamines and heightened systemic sympathetic activity [171, 172]. At the tissue level, however, a distinct pattern emerges: regenerating nodules in cirrhotic livers exhibit parenchymal denervation, whereas nerve fibers persist and even increase within portal tracts and fibrous septa [173, 174]. Thus, cirrhosis is characterized by parenchymal hypo‐innervation relative to hyper‐innervation of fibrotic regions, as detailed in Table 2.

**TABLE 2:** Autonomic nervous system dysregulation in cirrhosis and HCC.

Pathological process	Therapeutic targets/molecules	Drug or intervention	Animal/cell model	Mechanism	Outcome
Anatomical alterations					
Pre‐cirrhotic and established cirrhosis [173]	Autonomic nerve fibers	—	Human liver biopsies	Loss of parenchymal nerve fibers in regenerating nodules; nerve fibers preserved in fibrous septa	Parenchymal denervation in cirrhotic nodules
Chronic liver diseases (including cirrhotic stages) [242]	Nerve fibers	—	Human cirrhotic biopsies	Increased mast cell and nerve fiber densities in stroma; mast cell‐nerve contacts	Mast cell and fiber densities correlated with fibrosis severity (includes cirrhosis)
CCl4‐induced cirrhosis [239]	Cholinergic nerve fibers	—	Cirrhotic rats	Nerve fibers proliferated in fibrous septa and formed close contacts with mast cells and myofibroblasts	Formation of mast cell/myofibroblast/cholinergic nerve terminal complexes
HCC (human) [19]	α1‐AR	—	Human HCC tissues	High density of sympathetic nerve fibers in HCC tissues; α1‐AR expressed on KCs	High sympathetic nerve fiber density correlated with poor prognosis
HCC (human) [178]	β2‐AR, α7nAChR, M1 and M3 mAChRs	—	Human HCC tissues; human hepatoma cell lines; primary hepatoma cells	High density of sympathetic and parasympathetic nerve fibers in HCC tissues; overexpression of adrenergic and cholinergic receptors in hepatoma cells	High autonomic nerve fiber density correlated with poor prognosis
HCC (human) [179]	CHRM3, nAChRs	—	Human HCC tissues; human hepatoma cell lines	High density of cholinergic neural cells in HCC; CHRM3 overexpression	Cholinergic orientation associated with poor prognosis and aggressive HCC features
Functional role of sympathetic signaling					
CCl4‐induced chronic liver injury and cirrhosis [175]	Sympathetic nervous system	6‐OHDA (chemical sympathectomy); SHR (genetic sympathetic hyperactivity)	Rats; HepG2 cells	6‐OHDA ablated sympathetic nerves and reduced NE; SHR increased sympathetic activity and increased NE	Sympathetic hyperactivity worsened liver fibrosis and cirrhosis; sympathectomy attenuated liver injury and fibrosis
DEN‐induced HCC [19]	α1‐AR	6‐OHDA; prazosin (antagonist)	DEN‐treated rats; primary rat KCs	Sympathetic denervation or α1‐AR blockade reduced KC activation and IL‐6/TGF‐β secretion	Reduced HCC incidence and tumor development
HCC [180]	MAOA; α1‐AR; β2‐AR	Clorgyline (MAOA inhibitor); prazosin (α1‐AR antagonist); ICI118551 (β2‐AR antagonist)	Human HCC tissues; HCC cell lines; nude mouse metastasis models	MAOA degraded NE/Epi; NE/Epi activatedα1‐AR andβ2‐AR; AR transactivated EGFR via MMP7/ADAM12/HB‐EGF pathway	MAOA downregulation correlated with poor prognosis; MAOA overexpression or AR/EGFR inhibition suppressed HCC invasion and anoikis
HCC [181]	β1‐AR	β1‐AR signaling	Human HCC tissues; MAIT cells	β1‐AR signaling promoted FOXP3^+^CXCR3^+^ MAITreg induction and function; MAITregs inhibited antitumor immune responses	High intra‐tumoral MAITreg proportion correlated with poor clinical outcomes
DEN/CCl4‐induced HCC, transplantable HCC [182]	β‐AR	Enriched environment exposure	Mice; hepatocyte cell lines	Sympathetic/β‐AR/CCL2 axis activation; CD8^+^ T cell‐dependent mechanism	Reduced liver tumor incidence and growth; overcame PD‐L1 resistance; improved anti‐tumor immunity
Chronic stress‐driven HCC [183]	β2‐AR, QPRT	CUMS; 6‐OHDA; AAV8‐TBG‐β2‐AR /Qprt	Mice; human HCC tissues; human liver organoids	Chronic stress reduced catecholamines; downregulated hepatocyte β2‐AR; decreased cAMP‐CREB‐QPRT axis; impaired mitochondrial function of liver CD8^+^ T cells	Accelerated HCC progression; reduced CD8^+^ T cell number and function; NAM or β2‐AR /QPRT overexpression restored CD8^+^ T cell immunity and reduced HCC
Clinical features and therapy					
Cirrhosis (clinical) [176]	Autonomic nervous system	—	Cirrhotic patients	Reduced heart rate variability; impaired baroreflex sensitivity; abnormal CARTs	Autonomic neuropathy predicted higher mortality and cirrhosis‐related complications
Portal hypertension, variceal rebleeding, ascites, SBP, HRS, hepatic encephalopathy [177]	β‐AR	Propranolol (antagonist)	Cirrhotic patients	β‐AR blockade reduced hepatic venous pressure gradient	Reduced variceal rebleeding, ascites, SBP, HRS, encephalopathy; improved survival

**Abbreviations: **6‐OHDA, 6‐hydroxydopamine; ADAM12, A disintegrin and metalloproteinase 12; AR, adrenergic receptor; cAMP, cyclic adenosine monophosphate; CARTs, cardiovascular autonomic reflex tests; CCL2, chemokine (C‐C motif) ligand 2; CCl4, carbon tetrachloride; CHRM3, cholinergic receptor muscarinic 3; CREB, cAMP response element‐binding protein; CUMS, chronic unpredictable mild stress; DEN, diethylnitrosamine; EGFR, epidermal growth factor receptor; Epi, epinephrine; HB‐EGF, heparin‐binding epidermal growth factor‐like growth factor; HCC, hepatocellular carcinoma; HRS, hepatorenal syndrome; IL‐6, interleukin‐6; KC, Kupffer cell; mAChR, muscarinic acetylcholine receptor; MAIT, mucosal‐associated invariant T; MAOA, monoamine oxidase A; MMP7, matrix metalloproteinase 7; nAChRs, nicotinic acetylcholine receptor; NAM, nicotinamide; NE, norepinephrine; PD‐L1, programmed death‐ligand 1; QPRT, quinolinate phosphoribosyltransferase; SBP, spontaneous bacterial peritonitis; SHR, spontaneously hypertensive rat; TGF‐β, transforming growth factor‐β; α7nAChR, α7 Nicotinic acetylcholine receptor.

The functional role of sympathetic signaling in cirrhosis is complex, as detailed in Table 2. While NE can promote hepatocyte proliferation and protect against apoptosis [155], sympathetic activity also drives cirrhosis progression by accelerating hepatocyte growth kinetics following injury [175]. Clinically, cirrhosis is associated with autonomic neuropathy, manifested as reduced heart rate variability [176]. Notably, β‐adrenergic blockade with propranolol reduces portal pressure and improves survival in cirrhotic patients with variceal bleeding [177]. Whether this reflects direct modulation of hepatic sympathetic tone or systemic hemodynamic effects remains to be determined.

### 4.7 Dysregulation in HCC

The autonomic nervous system is a key modulator of HCC progression and metastasis, as detailed in Table 2. In human HCC tissues, elevated densities of sympathetic and parasympathetic nerve fibers correlate with poor prognosis [19, 178], and hepatoma cells overexpress β2‐adrenergic and cholinergic receptors [178]. Transcriptomic analyses further reveal a cholinergic polarity shift, with M3R enrichment associated with aggressive tumor features and shortened survival [179].

Sympathetic activation promotes hepatocarcinogenesis. In diethylnitrosamine‐induced HCC models, sympathetic denervation attenuates fibrosis and tumor development [19]. Mechanistically, sympathetic outflow engages α1‐AR on Kupffer cells, driving IL‐6 and transforming growth factor (TGF)‐β secretion to shape an immunosuppressive niche [19]. Additionally, downregulation of monoamine oxidase A, the enzyme responsible for NE degradation, is associated with enhanced vascular invasion, metastasis, and poor survival in HCC [180], suggesting that local NE accumulation may further fuel tumor progression. Recent single‐cell analyses have also identified a regulatory subset of mucosal‐associated invariant T cells in HCC, whose immunosuppressive function is potentiated by β1‐AR signaling [181].

Environmental and neuroendocrine influences further modulate HCC progression. Enriched environment exposure protects mice from liver tumors through a CD8^+^ T cell‐dependent sympathetic/β‐AR/CCL2 axis [182], highlights that sympathetic signaling can exert anti‐tumor effects depending on context. Moreover, a recent study by Sun et al. demonstrated that chronic stress drives HCC progression by disrupting a hypothalamus–liver axis. Specifically, stress‐induced catecholamine/β2‐AR signaling downregulates hepatocyte quinolinate phosphoribosyl transferase, causing kynurenine pathway imbalance and impairing liver CD8^+^ T cell immunity. This work provides direct evidence linking hypothalamic sympathetic output to hepatic metabolic reprogramming and immune surveillance in HCC [183]. Whether this pathway is broadly applicable across HCC etiologies remains to be explored.

### 4.8 Hypothalamic‐Pituitary Disorders and Surgeries in Liver Disease

Hypopituitarism, particularly GH deficiency, is strongly associated with hepatic steatosis and fibrosis. Patients with childhood‐onset hypopituitarism exhibit significantly higher MASLD prevalence than matched controls, with lower insulin‐like growth factor‐1 standard deviation score independently associated with both steatosis and fibrosis [184]. In patients with nonfunctioning pituitary adenomas, GH deficiency confers approximately twofold higher MASLD prevalence, independent of age, cholesterol, and gonadal function [185]. A pediatric case of GH deficiency progressing to cirrhosis at age 47 underscores that MASLD can progress to end‐stage liver disease in this population [186].

Beyond GH deficiency, HPT axis dysfunction has been recognized as an independent driver of liver disease [38], with central hypothyroidism prevalent in craniopharyngioma patients and significantly associated with cirrhosis [187]. Panhypopituitarism represents a more severe clinical model, with surgical etiologies associated with accelerated fibrosis compared to non‐surgical cases [188]. In one extreme example, an 8‐year‐old with panhypopituitarism from craniopharyngioma resection required liver transplantation for rapidly progressing metabolic dysfunction‐associated steatohepatitis (MASH) [189].

Craniopharyngioma survivors frequently develop hypothalamic obesity and metabolic syndrome due to structural hypothalamic damage, predisposing them to MASLD. In adult craniopharyngioma survivors, MASLD ranges from 62.5%–67.4%, with advanced fibrosis in 6.3%–32.5% [190, 191]. Direct evidence linking sellar surgery to accelerated fibrosis comes from a comparative study showing significantly more advanced hepatic fibrosis in surgical versus non‐surgical hypopituitarism patients, suggesting that surgical intervention itself may contribute to disease progression [188].

In contrast, Cushing's syndrome exemplifies chronic HPA axis hyperactivity. Hepatic steatosis is present in 26.5% of patients at diagnosis, with resolution after cortisol normalization [192]. MASLD prevalence in Cushing's syndrome (66.1%) significantly exceeds that in patients with nonfunctional adrenal incidentalomas (26.2%) [193], and lower free thyroxine levels independently predict MASLD in active Cushing's disease [194].

Collectively, these clinical observations validate that hypothalamic‐pituitary axis disruption—whether primary (Cushing's syndrome, GH deficiency) or secondary to tumor and surgery (craniopharyngioma, post‐surgical hypothalamic damage)—is sufficient to drive progressive liver disease. From a nosological perspective, steatotic liver disease secondary to hypothalamic dysfunction is best classified as a specific form of secondary steatotic liver disease [195], although clinical overlap with MASLD is common.

## 5 Therapeutic Horizons: Targeting the Hypothalamus‐Liver Axis

**FIGURE 6:**
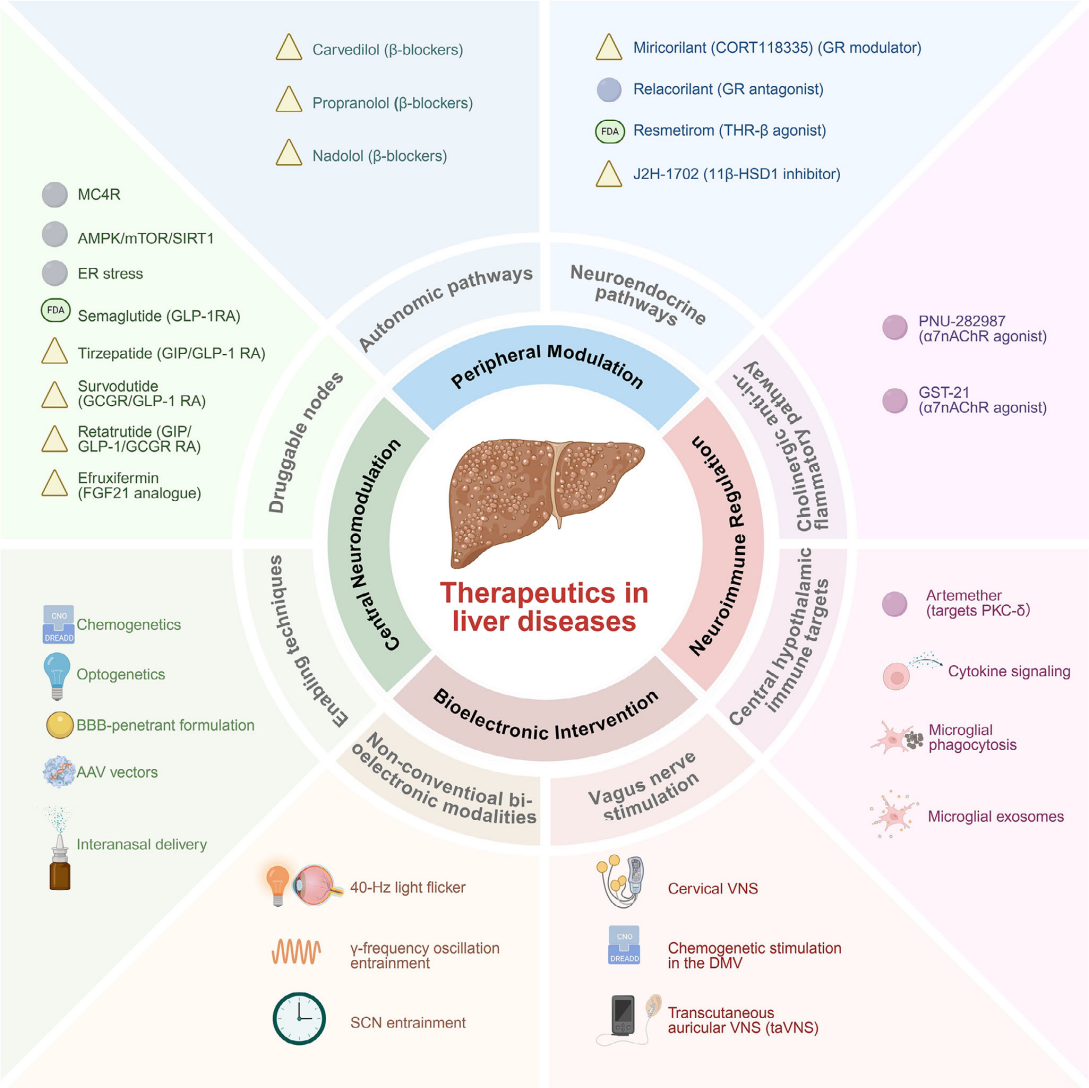
Therapeutic strategies targeting the hypothalamus‐liver axis for liver diseases. Overview of four therapeutic modalities: circuit‐specific pharmacology, peripheral effector targeting, neuroimmune modulation, and bioelectronic intervention. 11β‐HSD1, 11β‐hydroxysteroid dehydrogenase type 1; AAV, adeno‐associated virus; AMPK, AMP‐activated protein kinase; BBB, blood brain barrier; DMV, dorsal motor nucleus of the vagus; ER, endoplasmic reticulum; FGF21, fibroblast growth factor 21; GCGR, glucagon receptor; GIP, glucose‐dependent insulinotropic polypeptide; GLP‐1, glucagon‐like peptide‐1; GR, glucocorticoid receptor; MC4R, Melanocortin 4 Receptor; mTOR, mammalian target of rapamycin; SIRT1, sirtuin 1; PKC‐δ, Protein kinase C δ; SCN, suprachiasmatic nucleus; THR‐β, thyroid hormone receptor β; VNS, vagus nerve stimulation. Created in BioRender. Tang, Q. (2026) https://BioRender.com/bnfaatr.

Despite recent therapeutic breakthroughs for liver diseases, effective management remains challenging. Targeting the hypothalamus‐liver axis has emerged as a key frontier for transformative treatments, with four complementary strategies: direct modulation of hypothalamic circuits, targeting peripheral effector pathways, neuroimmune modulation, and bioelectronic intervention (Figure 6).

First, direct modulation of hypothalamic circuits represents a central strategy. Preclinical studies using chemogenetics and optogenetics have established causal links between hypothalamic nuclei and hepatic functions [49, 76]. Mechanistic studies have identified druggable molecular targets within these circuits, such as MC4R and nutrient‐sensing kinases (AMPK, mTOR, SIRT1) [196, 197]. Emerging evidence further implicates hypothalamic ER stress as a critical integrator, where its selective inhibition in the PVN ameliorates obesity‐related hepatic steatosis [198]. Clinically, the GLP‐1 receptor agonist semaglutide received FDA approval in August 2025 for MASH with moderate‐to‐advanced fibrosis based on the phase 3 ESSENCE trial [199, 200]. In the trial, semaglutide showed a 62.9% vs 34.3% improvement in steatohepatitis resolution (placebo‐adjusted difference, 28.7%) and a 36.8% vs 22.4% improvement in fibrosis without worsening of MASH (placebo‐adjusted difference, 14.4%) [199]. Semaglutide acts primarily through central GLP‐1 receptor activation in the ARC, modulating POMC and AgRP neuronal activity to reduce food intake and promote weight loss, rather than through direct hepatocyte GLP‐1 receptor signaling [201]. Beyond GLP‐1 receptor agonists (GLP‐1RAs), the dual glucose‐dependent insulinotropic polypeptide (GIP)/GLP‐1RA tirzepatide has recently demonstrated efficacy in reducing major adverse liver outcomes, including cirrhosis [202], while other multi‐receptor agonists (e.g., survodutide [glucagon receptor (GCGR)/GLP‐1], retatrutide [GIP/GLP‐1/GCGR]) show promise for hepatic fat reduction [203, 204]. Furthermore, FGF21 analogues (e.g., efruxifermin) have shown anti‐fibrotic efficacy [205]. While challenges remain in achieving circuit‐specific delivery, advances in BBB‐penetrant formulations, adeno‐associated virus vectors, and intranasal delivery offer promising avenues for precise neuromodulation [206, 207].

Second, targeting peripheral effector pathways offers a complementary strategy. For sympathetic overactivity, non‐selective β‐blockers (carvedilol, propranolol, nadolol) are established for managing portal hypertension in cirrhosis [208]. For the parasympathetic arm, the vagus nerve exerts anti‐inflammatory effects via the α7nAChR pathway [209]. Targeting the HPA axis, selective GR modulators have shown promise. For example, miricorilant (CORT118335) has demonstrated preliminary efficacy in reducing hepatic lipid accumulation in mouse models of MASLD [210], and is currently under phase II investigation for MASH [211]. Relacorilant, a highly selective GR antagonist, improved liver function tests in Cushing's syndrome patients [212]. Complementary to these strategies, directly targeting the terminal hepatic effectors bypasses central feedback loops. The THR‐β agonist resmetirom activates hepatic THR‐β without altering central HPT axis activity [213], leading to its regulatory approval for MASH [214, 215]. Other liver‐directed agents, such as the 11β‐HSD1 inhibitor J2H‐1702, have also shown preliminary efficacy in reducing hepatic fat content [216]. However, the stage‐dependent efficacy of liver‐directed agents across the MASLD spectrum and the long‐term safety of GR modulation remain to be fully defined.

Third, neuroimmune modulation targeting the cholinergic anti‐inflammatory pathway and hypothalamic immune integration centers offers a pharmacologically tractable strategy [9]. In the peripheral vagus‐based reflex, afferent vagal fibers sense inflammation, relay signals to the hypothalamus, and activate efferent vagal cholinergic neurons to release ACh, which acts on α7nAChRs on Kupffer cells to inhibit cytokine production [9]. Preclinical validation has been achieved using selective α7nAChR agonists. PNU‐282987 protects against acute liver injury [217], while GST‐21 shows hepatoprotective effects in sepsis‐related liver injury [218]. However, target specificity remains a concern due to the widespread expression of α7nAChR. The antimalarial drug artemether improves hepatic lipid metabolism by directly targeting PKC‐δ in hypothalamic microglia [219], although its mechanism in humans and translational potential is uncertain. Beyond specific drug candidates, mechanistic studies have revealed additional conceptional nodes for therapeutic intervention, including cytokine‐mediated neuroimmune signaling, phagocytosis‐dependent remodeling of hypothalamic circuits, and exosome‐mediated delivery of neuroinflammatory signals from microglia to the liver [220]. Clinical validation, however, is still lacking for most of these targets.

Finally, bioelectronic intervention directly restores autonomic balance within the hypothalamus‐liver axis [221]. Preclinical studies show that cervical vagus nerve stimulation (VNS) reduces hepatic steatosis in MASLD models [222], and chemogenetic activation of DMV parasympathetic neurons reduces hepatic lipid accumulation in a MASH mouse model [223]. Clinically, transcutaneous auricular VNS (taVNS) in a randomized controlled trial of 30 metabolic syndrome patients improved heart rate variability parameters and reduced systolic blood pressure by 8.3 mmHg after 8 weeks [224]. Reduced heart rate variability parameters independently predict MASLD risk and correlate with disease severity [225]. In a mouse model of ethanol‐induced hepatic steatosis, 40‐Hz light flicker reduced hepatic lipid accumulation and restored circadian clock proteins in the SCN, demonstrating that γ‐frequency oscillation‐mediated SCN entrainment underlies its circadian restorative effects [226]. However, optimal stimulation parameters and stage‐dependent efficacy remain to be determined, and larger clinical trials are needed.

## 6 Challenges and Future Directions

The recognition of the hypothalamus–liver axis as a therapeutic target represents a pivotal conceptual advance. However, translating this insight into clinical applications requires overcoming fundamental knowledge gaps and technological limitations.

A key conceptual challenge lies in distinguishing cause from consequence in neural remodeling observed in liver diseases. It remains unclear whether pathological changes in hypothalamic circuits and hepatic innervation actively drive disease progression or merely reflect secondary adaptations. This evolving relationship creates dynamic therapeutic targets that necessitate stage‐specific intervention strategies and raises concerns about disease‐related neuroplasticity altering therapeutic responsiveness over time.

Technological limitations further impede progress. The absence of non‐invasive, organ‐selective autonomic biomarkers restricts mechanistic investigation and clinical monitoring. Candidate biomarkers for patient stratification—including heart rate variability, plasma catecholamine levels, and HPA axis status—remain to be validated. Neuroimaging modalities have not yet been integrated into clinical workflows for liver diseases. Moreover, existing neuro‐modulatory approaches lack precision in targeting hepatic autonomic signaling without systemic effects, and stimulation parameter optimization remains empirical, though machine learning may offer adaptive solutions. Additionally, sex differences in hypothalamic‐liver axis regulation remain underexplored.

Future research should prioritize establishing causal relationships using advanced genetic tools. A critical objective is to elucidate how specific hypothalamic neuronal populations communicate with distinct hepatic cell types and to identify stage‐specific regulatory nodes. Concurrently, developing validated non‐invasive biomarkers will be essential for patient stratification and treatment monitoring. Future research should also prioritize sex‐specific analyses to elucidate potential dimorphisms in hypothalamus‐liver circuit function and therapeutic responses.

A specific unresolved question concerns the physiological consequences of vagus nerve transection during metabolic and bariatric surgery (MBS). While MBS effectively resolves hepatic steatosis and improves metabolic parameters through substantial and sustained weight loss, fibrosis regression is not always observed [227], suggesting that mechanisms beyond weight loss, including potential vagal contributions, may be involved. The independent role of vagotomy remains debated; rodent studies show paradoxical effects on steatosis [25, 228, 229], and clinical data on its metabolic impact are inconsistent [230]. Notably, MBS is associated with an increased risk of alcohol use disorder and ALD [231], potentially through vagal damage to reward circuitry and HPA axis regulation [232, 233]. Whether vagotomy independently contributes to these effects remains unclear. Elucidating these mechanisms represents a critical area for future investigation, as the vagus nerve‐liver axis offers a unique human model to understand parasympathetic regulation of hepatic metabolism.

Beyond these specific unresolved questions, interventions must account for disease progression in a broader translational context, as strategies effective in early metabolic dysfunction may prove inadequate in advanced stages. Rigorous validation in human‐relevant systems is essential. Given the multifactorial pathogenesis, rationally designed combination therapies that simultaneously target neural circuits and peripheral effector mechanisms will likely be necessary to achieve durable therapeutic responses.

## 7 Conclusions and Perspectives

This review establishes the hypothalamic‐liver axis as a central command hub in the hierarchical control of systemic homeostasis. It functions as a sophisticated multilayer integration network that processes various signals to precisely govern hepatic glucose and lipid homeostasis, bile acid metabolism, and regenerative capacity. The physiological function of this axis relies fundamentally on a dynamic, balanced interplay between autonomic and neuroendocrine pathways.

Dysregulation of this finely tuned communication system has now been convincingly linked to the pathogenesis of a broad disease spectrum, from steatotic liver disease to HCC. Decoding the axis is a critical frontier in confronting the global burden of metabolic and liver diseases. A detailed functional map of its neural circuits, signaling mechanisms, and cell‐type‐specific effects will form the essential foundation for a new therapeutic paradigm centered on bioelectronic and neuro‐modulatory medicine. This approach aims to shift from managing downstream symptoms toward directly recalibrating the core regulatory system, offering a transformative strategy for metabolic health, albeit with major knowledge gaps that remain to be addressed.

Moving forward, the field must advance in parallel on several fronts: elucidating circuit‐level mechanisms, validating molecular targets, and developing targeted delivery systems, including brain‐penetrant drugs, viral vectors, and non‐invasive neuromodulation devices. The integration of cutting‐edge tools, such as multi‐organ spatial multi‐omics, “multi‐tissue” organoid models, optogenetics, and single‐cell transcriptomics, will be essential to deconstruct the neuro‐metabolic crosstalk within the brain‐liver axis at the whole‐body level. Ultimately, synthesizing insights across neuroscience, systems immunology, and bioengineering will translate our growing mechanistic knowledge into rationally designed, precise, and combination therapies, transforming the therapeutic landscape for patients with liver diseases.

### 7.1 Search Strategy and Selection Criteria

The literature cited in this Review was identified through the following search strategy. We performed searches in PubMed using keywords relevant to each section (e.g., “hypothalamus–liver axis”, “hepatic autonomic innervation”, “hypothalamic or brain regulation of hepatic glucose, lipid, and bile acid metabolism”, “liver regeneration”, etc.). Retrieved articles were manually screened for relevance. Priority was given to mechanistic studies and recent high‐impact reviews published within the past decade, with seminal older works included where appropriate.

## Author Contributions

Q.T., L.M., and J.H. contributed to the conception and design of the manuscript. Q.T. drafted the manuscript. Q.T., J.L., and H.S. drew the figures and summarized the tables. A.Z., H.Z., Y.X., L.M., and J.H. contributed to the critical revisions of the manuscript. All authors have read and approved the final manuscript.

## Conflicts of Interest

The authors declare no conflicts of interest.

## Data Availability

Data sharing not applicable to this article as no datasets were generated or analysed during the current study.
